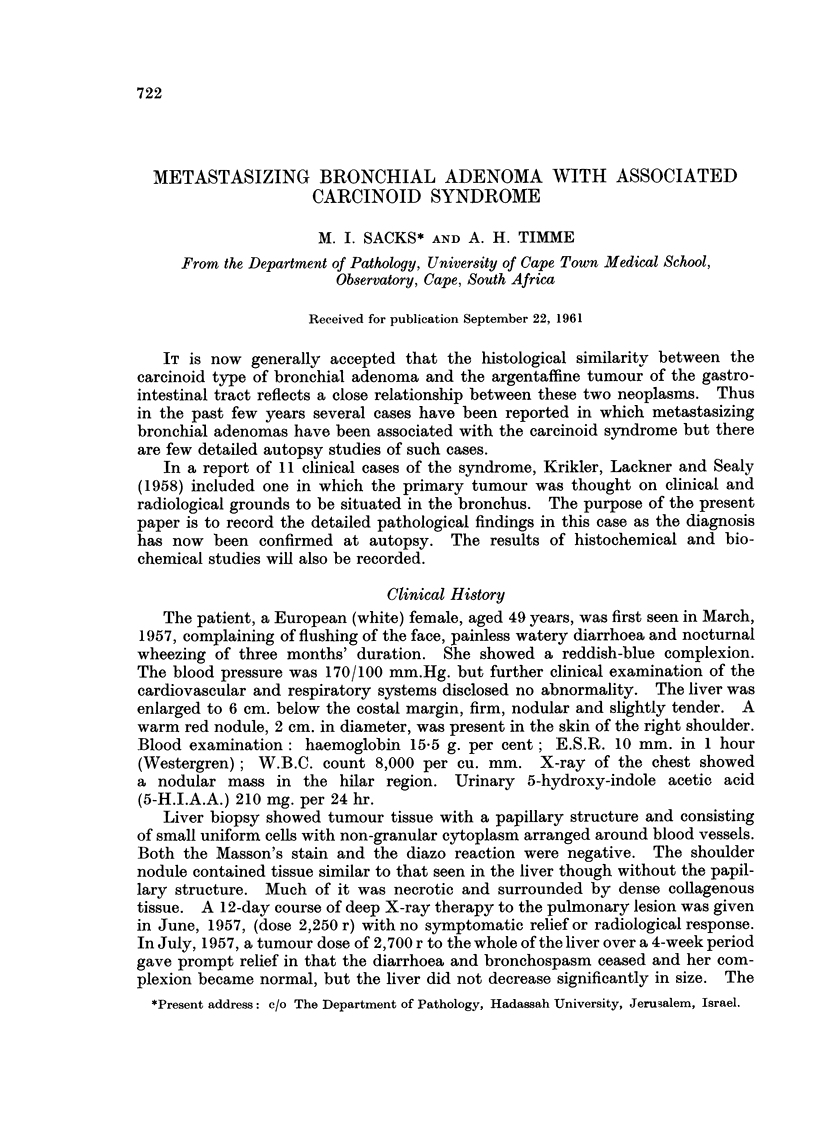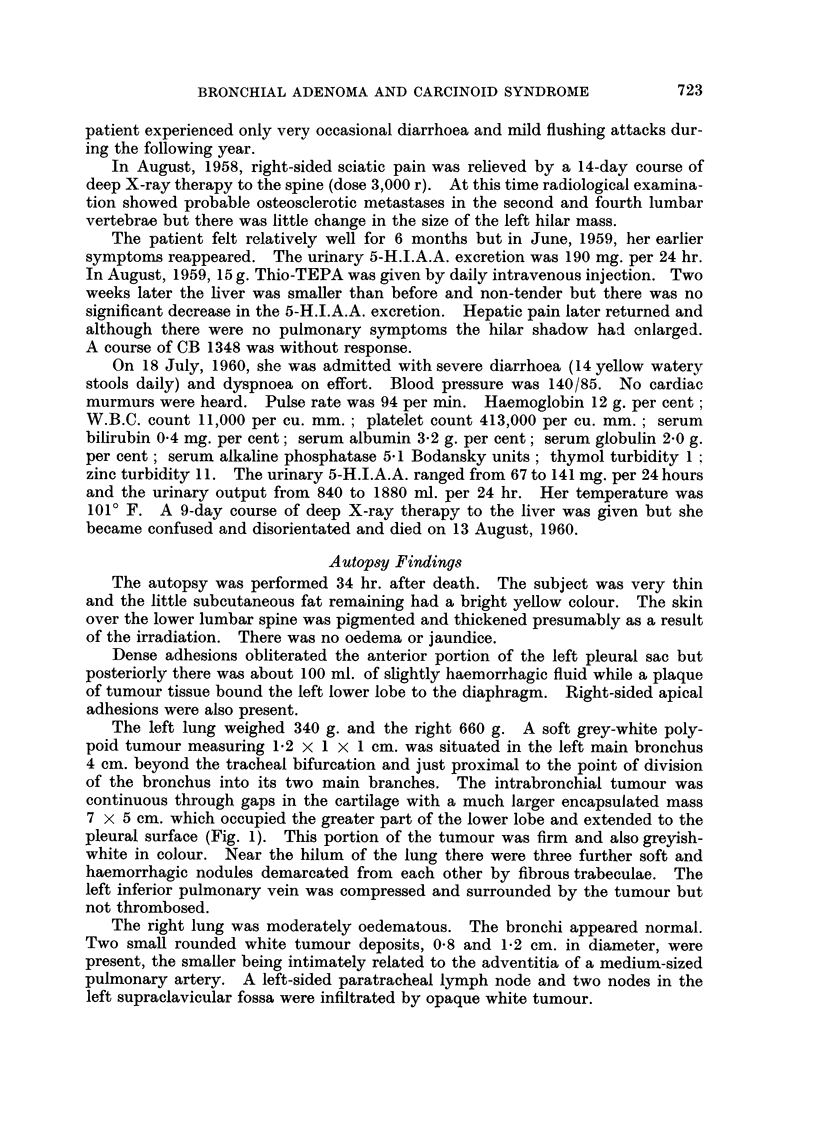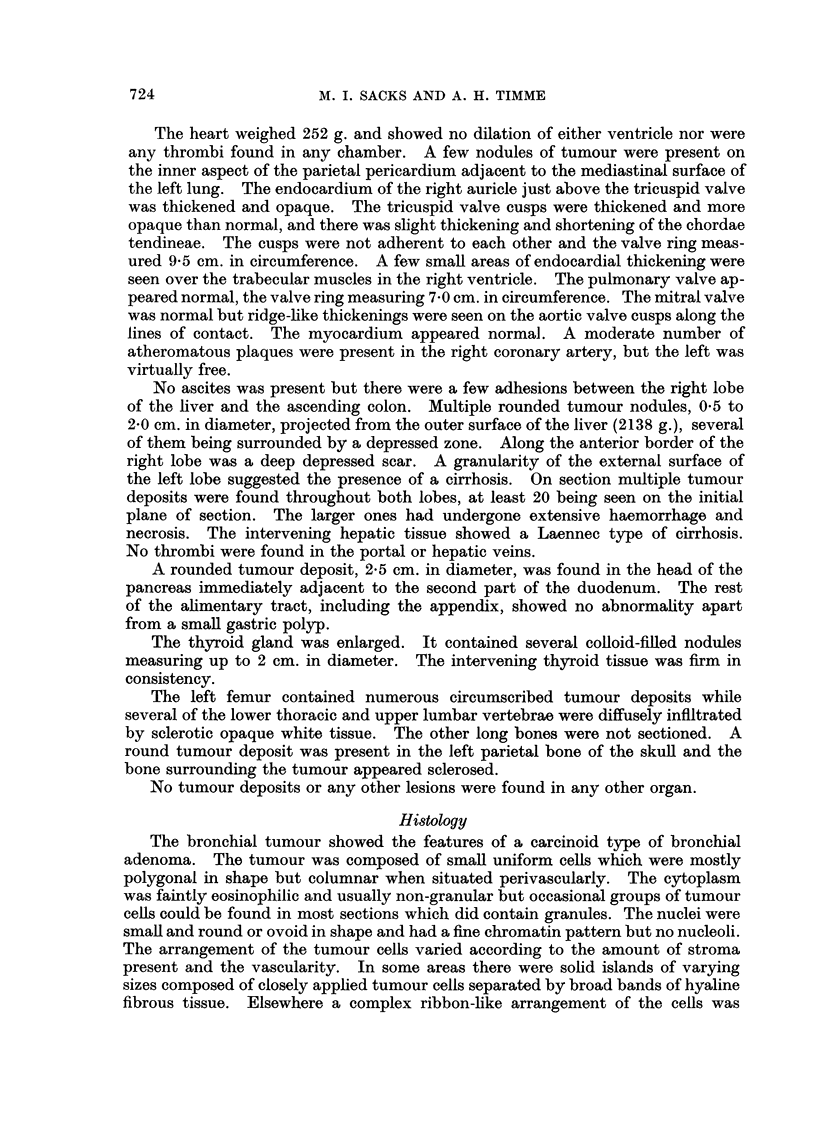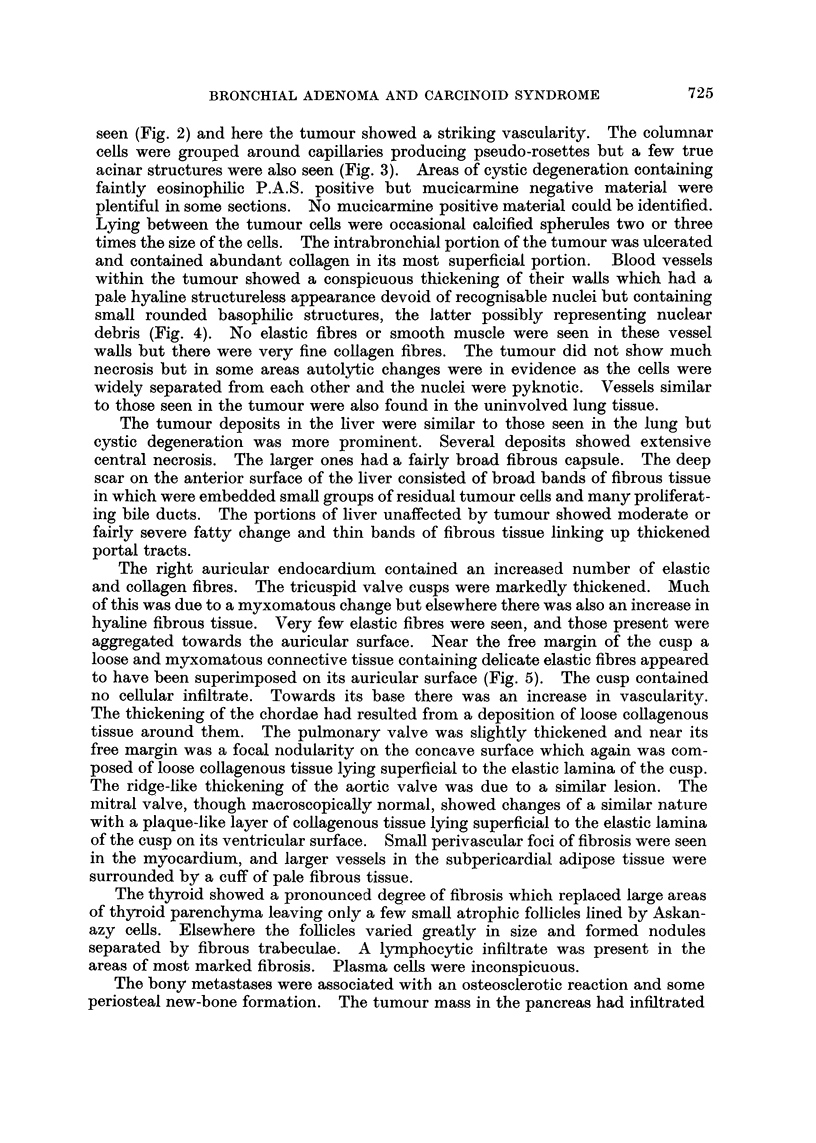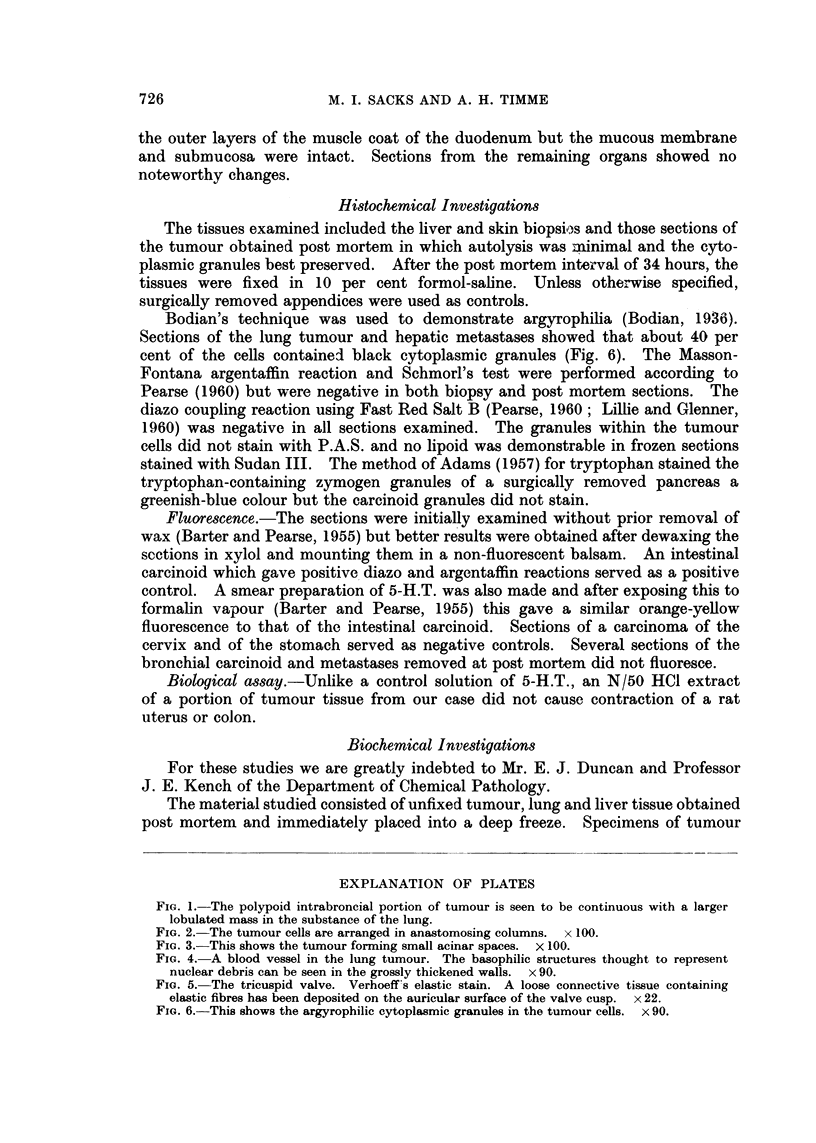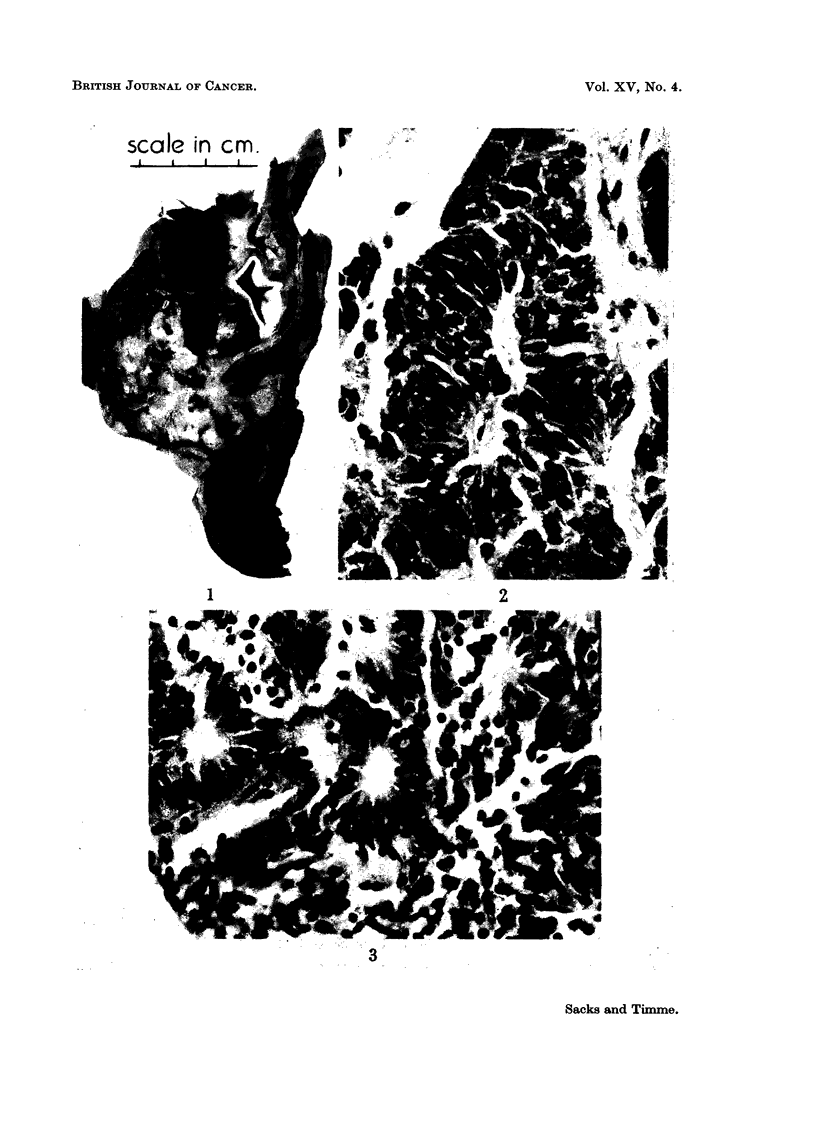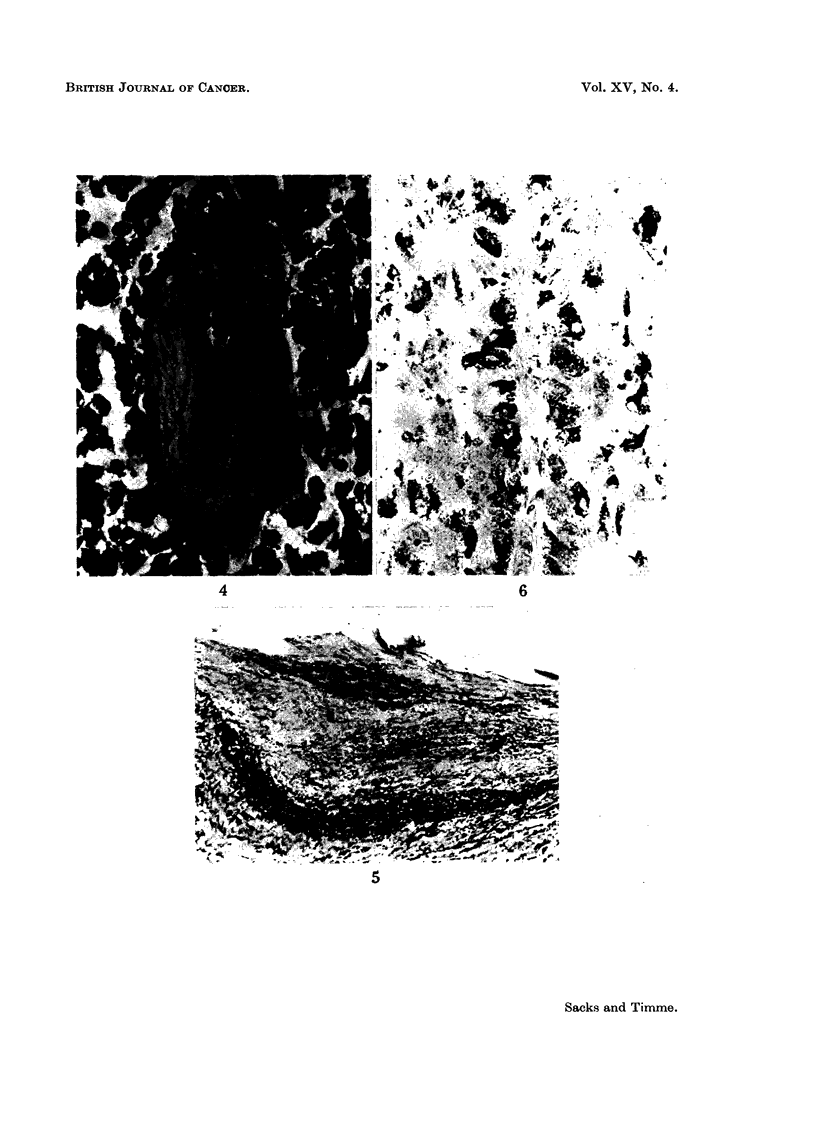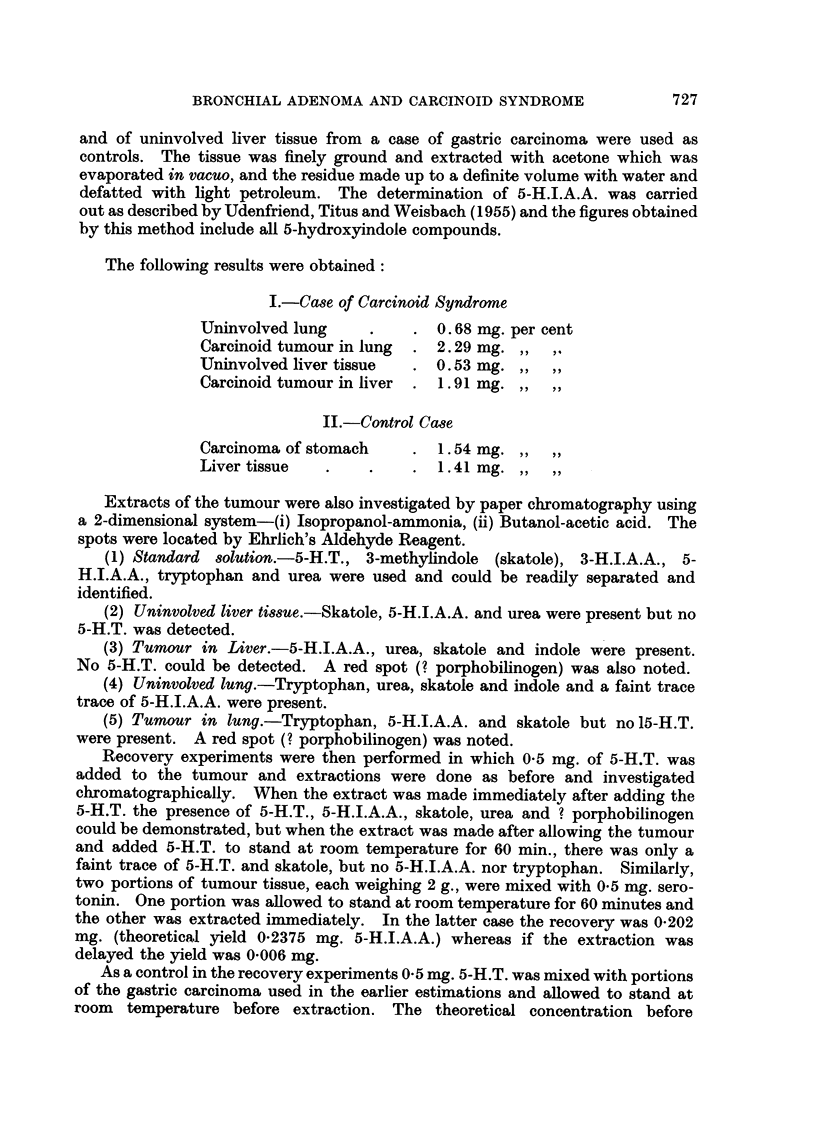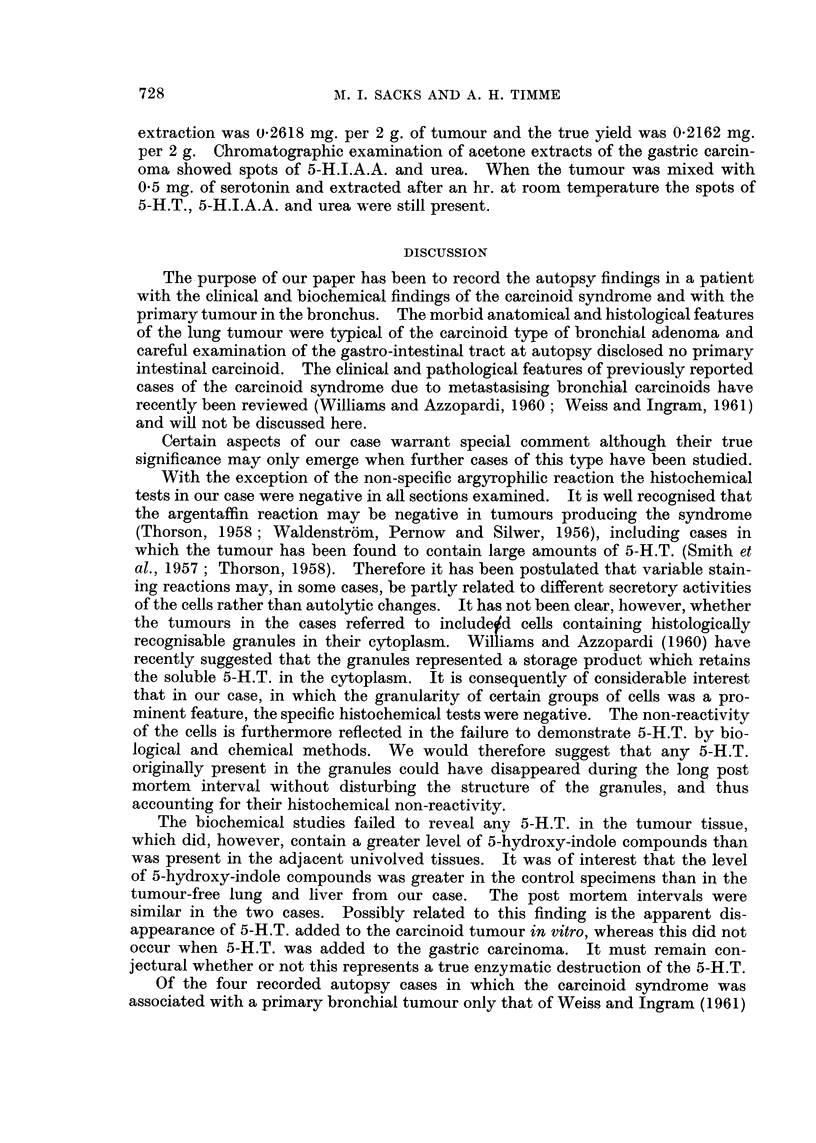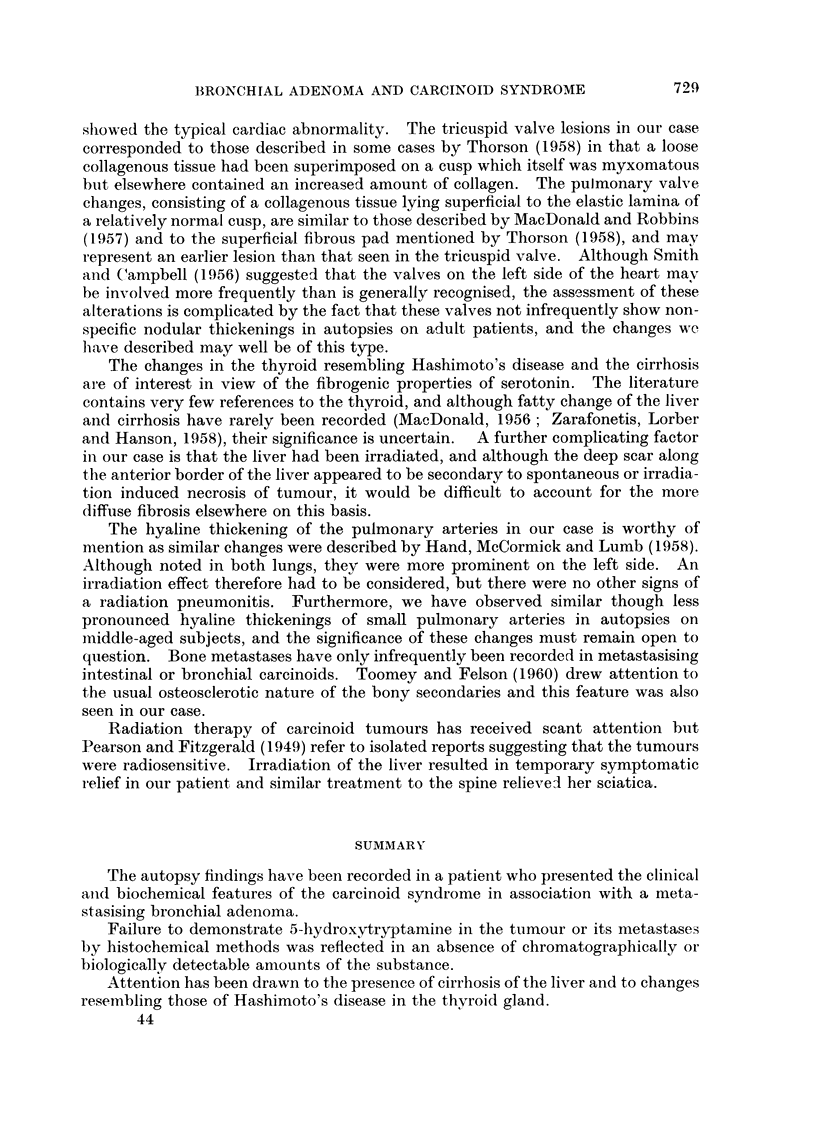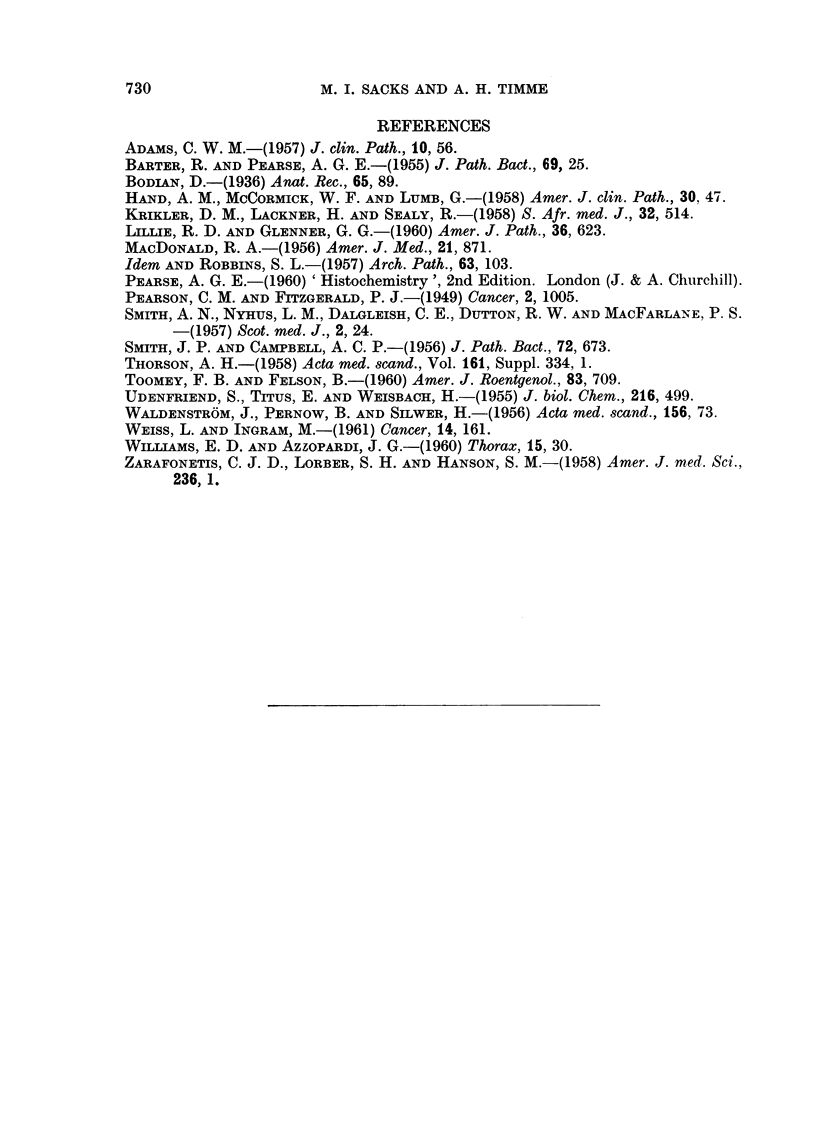# Metastasizing Bronchial Adenoma with Associated Carcinoid Syndrome

**DOI:** 10.1038/bjc.1961.82

**Published:** 1961-12

**Authors:** M. I. Sacks, A. H. Timme

## Abstract

**Images:**


### METASTASIZING BRONCHIAL ADENOMA WITH ASSOCIATED CARCINOID SYNDROME

M. I. SACKS* AND A. H. TIMME

*From the Department of Pathology, University of Cape Town Medical School, Observatory, Cape, South Africa*

Received for publication September 22, 1961

IT is now generally accepted that the histological similarity between the carcinoid type of bronchial adenoma and the argentaffine tumour of the gastro-intestinal tract reflects a close relationship between these two neoplasms. Thus in the past few years several cases have been reported in which metastasizing bronchial adenomas have been associated with the carcinoid syndrome but there are few detailed autopsy studies of such cases.

In a report of 11 clinical cases of the syndrome, Krikler, Lackner and Sealy (1958) included one in which the primary tumour was thought on clinical and radiological grounds to be situated in the bronchus. The purpose of the present paper is to record the detailed pathological findings in this case as the diagnosis has now been confirmed at autopsy. The results of histochemical and bio-chemical studies will also be recorded.

### *Clinical History*

The patient, a European (white) female, aged 49 years, was first seen in March, 1957, complaining of flushing of the face, painless watery diarrhoea and nocturnal wheezing of three months' duration. She showed a reddish-blue complexion. The blood pressure was 170/100 mm.Hg. but further clinical examination of the cardiovascular and respiratory systems disclosed no abnormality. The liver was enlarged to 6 cm. below the costal margin, firm, nodular and slightly tender. A warm red nodule, 2 cm. in diameter, was present in the skin of the right shoulder. Blood examination : haemoglobin 15·5 g. per cent ; E.S.R. 10 mm. in 1 hour (Westergren) ; W.B.C. count 8,000 per cu. mm. X-ray of the chest showed a nodular mass in the hilar region. Urinary 5-hydroxy-indole acetic acid (5-H.I.A.A.) 210 mg. per 24 hr.

Liver biopsy showed tumour tissue with a papillary structure and consisting of small uniform cells with non-granular cytoplasm arranged around blood vessels. Both the Masson's stain and the diazo reaction were negative. The shoulder nodule contained tissue similar to that seen in the liver though without the papillary structure. Much of it was necrotic and surrounded by dense collagenous tissue. A 12-day course of deep X-ray therapy to the pulmonary lesion was given in June, 1957, (dose 2,250 r) with no symptomatic relief or radiological response. In July, 1957, a tumour dose of 2,700 r to the whole of the liver over a 4-week period gave prompt relief in that the diarrhoea and bronchospasm ceased and her complexion became normal, but the liver did not decrease significantly in size. The

*Present address : c/o The Department of Pathology, Hadassah University, Jerusalem, Israel.

patient experienced only very occasional diarrhoea and mild flushing attacks during the following year.

In August, 1958, right-sided sciatic pain was relieved by a 14-day course of deep X-ray therapy to the spine (dose 3,000 r). At this time radiological examination showed probable osteosclerotic metastases in the second and fourth lumbar vertebrae but there was little change in the size of the left hilar mass.

The patient felt relatively well for 6 months but in June, 1959, her earlier symptoms reappeared. The urinary 5-H.I.A.A. excretion was 190 mg. per 24 hr. In August, 1959, 15 g. Thio-TEPA was given by daily intravenous injection. Two weeks later the liver was smaller than before and non-tender but there was no significant decrease in the 5-H.I.A.A. excretion. Hepatic pain later returned and although there were no pulmonary symptoms the hilar shadow had enlarged. A course of CB 1348 was without response.

On 18 July, 1960, she was admitted with severe diarrhoea (14 yellow watery stools daily) and dyspnoea on effort. Blood pressure was 140/85. No cardiac murmurs were heard. Pulse rate was 94 per min. Haemoglobin 12 g. per cent ; W.B.C. count 11,000 per cu. mm. ; platelet count 413,000 per cu. mm. ; serum bilirubin 0·4 mg. per cent ; serum albumin 3·2 g. per cent ; serum globulin 2·0 g. per cent ; serum alkaline phosphatase 5·1 Bodansky units ; thymol turbidity 1 ; zinc turbidity 11. The urinary 5-H.I.A.A. ranged from 67 to 141 mg. per 24 hours and the urinary output from 840 to 1880 ml. per 24 hr. Her temperature was 101° F. A 9-day course of deep X-ray therapy to the liver was given but she became confused and disorientated and died on 13 August, 1960.

### *Autopsy Findings*

The autopsy was performed 34 hr. after death. The subject was very thin and the little subcutaneous fat remaining had a bright yellow colour. The skin over the lower lumbar spine was pigmented and thickened presumably as a result of the irradiation. There was no oedema or jaundice.

Dense adhesions obliterated the anterior portion of the left pleural sac but posteriorly there was about 100 ml. of slightly haemorrhagic fluid while a plaque of tumour tissue bound the left lower lobe to the diaphragm. Right-sided apical adhesions were also present.

The left lung weighed 340 g. and the right 660 g. A soft grey-white polypoid tumour measuring 1·2 × 1 × 1 cm. was situated in the left main bronchus 4 cm. beyond the tracheal bifurcation and just proximal to the point of division of the bronchus into its two main branches. The intrabronchial tumour was continuous through gaps in the cartilage with a much larger encapsulated mass 7 × 5 cm. which occupied the greater part of the lower lobe and extended to the pleural surface (Fig. 1). This portion of the tumour was firm and also greyish-white in colour. Near the hilum of the lung there were three further soft and haemorrhagic nodules demarcated from each other by fibrous trabeculae. The left inferior pulmonary vein was compressed and surrounded by the tumour but not thrombosed.

The right lung was moderately oedematous. The bronchi appeared normal. Two small rounded white tumour deposits, 0·8 and 1·2 cm. in diameter, were present, the smaller being intimately related to the adventitia of a medium-sized pulmonary artery. A left-sided paratracheal lymph node and two nodes in the left supraclavicular fossa were infiltrated by opaque white tumour.

The heart weighed 252 g. and showed no dilation of either ventricle nor were any thrombi found in any chamber. A few nodules of tumour were present on the inner aspect of the parietal pericardium adjacent to the mediastinal surface of the left lung. The endocardium of the right auricle just above the tricuspid valve was thickened and opaque. The tricuspid valve cusps were thickened and more opaque than normal, and there was slight thickening and shortening of the chordae tendineae. The cusps were not adherent to each other and the valve ring measured 9·5 cm. in circumference. A few small areas of endocardial thickening were seen over the trabecular muscles in the right ventricle. The pulmonary valve appeared normal, the valve ring measuring 7·0 cm. in circumference. The mitral valve was normal but ridge-like thickenings were seen on the aortic valve cusps along the lines of contact. The myocardium appeared normal. A moderate number of atheromatous plaques were present in the right coronary artery, but the left was virtually free.

No ascites was present but there were a few adhesions between the right lobe of the liver and the ascending colon. Multiple rounded tumour nodules, 0·5 to 2·0 cm. in diameter, projected from the outer surface of the liver (2138 g.), several of them being surrounded by a depressed zone. Along the anterior border of the right lobe was a deep depressed scar. A granularity of the external surface of the left lobe suggested the presence of a cirrhosis. On section multiple tumour deposits were found throughout both lobes, at least 20 being seen on the initial plane of section. The larger ones had undergone extensive haemorrhage and necrosis. The intervening hepatic tissue showed a Laennec type of cirrhosis. No thrombi were found in the portal or hepatic veins.

A rounded tumour deposit, 2·5 cm. in diameter, was found in the head of the pancreas immediately adjacent to the second part of the duodenum. The rest of the alimentary tract, including the appendix, showed no abnormality apart from a small gastric polyp.

The thyroid gland was enlarged. It contained several colloid-filled nodules measuring up to 2 cm. in diameter. The intervening thyroid tissue was firm in consistency.

The left femur contained numerous circumscribed tumour deposits while several of the lower thoracic and upper lumbar vertebrae were diffusely infiltrated by sclerotic opaque white tissue. The other long bones were not sectioned. A round tumour deposit was present in the left parietal bone of the skull and the bone surrounding the tumour appeared sclerosed.

No tumour deposits or any other lesions were found in any other organ.

### *Histology*

The bronchial tumour showed the features of a carcinoid type of bronchial adenoma. The tumour was composed of small uniform cells which were mostly polygonal in shape but columnar when situated perivascularly. The cytoplasm was faintly eosinophilic and usually non-granular but occasional groups of tumour cells could be found in most sections which did contain granules. The nuclei were small and round or ovoid in shape and had a fine chromatin pattern but no nucleoli. The arrangement of the tumour cells varied according to the amount of stroma present and the vascularity. In some areas there were solid islands of varying sizes composed of closely applied tumour cells separated by broad bands of hyaline fibrous tissue. Elsewhere a complex ribbon-like arrangement of the cells was seen (Fig. 2) and here the tumour showed a striking vascularity. The columnar cells were grouped around capillaries producing pseudo-rosettes but a few true acinar structures were also seen (Fig. 3). Areas of cystic degeneration containing faintly eosinophilic P.A.S. positive but mucicarmine negative material were plentiful in some sections. No mucicarmine positive material could be identified. Lying between the tumour cells were occasional calcified spherules two or three times the size of the cells. The intrabronchial portion of the tumour was ulcerated and contained abundant collagen in its most superficial portion. Blood vessels within the tumour showed a conspicuous thickening of their walls which had a pale hyaline structureless appearance devoid of recognisable nuclei but containing small rounded basophilic structures, the latter possibly representing nuclear debris (Fig. 4). No elastic fibres or smooth muscle were seen in these vessel walls but there were very fine collagen fibres. The tumour did not show much necrosis but in some areas autolytic changes were in evidence as the cells were widely separated from each other and the nuclei were pyknotic. Vessels similar to those seen in the tumour were also found in the uninvolved lung tissue.

The tumour deposits in the liver were similar to those seen in the lung but cystic degeneration was more prominent. Several deposits showed extensive central necrosis. The larger ones had a fairly broad fibrous capsule. The deep scar on the anterior surface of the liver consisted of broad bands of fibrous tissue in which were embedded small groups of residual tumour cells and many proliferating bile ducts. The portions of liver unaffected by tumour showed moderate or fairly severe fatty change and thin bands of fibrous tissue linking up thickened portal tracts.

The right auricular endocardium contained an increased number of elastic and collagen fibres. The tricuspid valve cusps were markedly thickened. Much of this was due to a myxomatous change but elsewhere there was also an increase in hyaline fibrous tissue. Very few elastic fibres were seen, and those present were aggregated towards the auricular surface. Near the free margin of the cusp a loose and myxomatous connective tissue containing delicate elastic fibres appeared to have been superimposed on its auricular surface (Fig. 5). The cusp contained no cellular infiltrate. Towards its base there was an increase in vascularity. The thickening of the chordae had resulted from a deposition of loose collagenous tissue around them. The pulmonary valve was slightly thickened and near its free margin was a focal nodularity on the concave surface which again was composed of loose collagenous tissue lying superficial to the elastic lamina of the cusp. The ridge-like thickening of the aortic valve was due to a similar lesion. The mitral valve, though macroscopically normal, showed changes of a similar nature with a plaque-like layer of collagenous tissue lying superficial to the elastic lamina of the cusp on its ventricular surface. Small perivascular foci of fibrosis were seen in the myocardium, and larger vessels in the subpericardial adipose tissue were surrounded by a cuff of pale fibrous tissue.

The thyroid showed a pronounced degree of fibrosis which replaced large areas of thyroid parenchyma leaving only a few small atrophic follicles lined by Askanazy cells. Elsewhere the follicles varied greatly in size and formed nodules separated by fibrous trabeculae. A lymphocytic infiltrate was present in the areas of most marked fibrosis. Plasma cells were inconspicuous.

The bony metastases were associated with an osteosclerotic reaction and some periosteal new-bone formation. The tumour mass in the pancreas had infiltrated the outer layers of the muscle coat of the duodenum but the mucous membrane and submucosa were intact. Sections from the remaining organs showed no noteworthy changes.

### *Histochemical Investigations*

The tissues examined included the liver and skin biopsies and those sections of the tumour obtained post mortem in which autolysis was minimal and the cytoplasmic granules best preserved. After the post mortem interval of 34 hours, the tissues were fixed in 10 per cent formol-saline. Unless otherwise specified, surgically removed appendices were used as controls.

Bodian's technique was used to demonstrate argyrophilia (Bodian, 1936). Sections of the lung tumour and hepatic metastases showed that about 40 per cent of the cells contained black cytoplasmic granules (Fig. 6). The Masson-Fontana argentaffin reaction and Schmorl's test were performed according to Pearse (1960) but were negative in both biopsy and post mortem sections. The diazo coupling reaction using Fast Red Salt B (Pearse, 1960 ; Lillie and Glenner, 1960) was negative in all sections examined. The granules within the tumour cells did not stain with P.A.S. and no lipoid was demonstrable in frozen sections stained with Sudan III. The method of Adams (1957) for tryptophan stained the tryptophan-containing zymogen granules of a surgically removed pancreas a greenish-blue colour but the carcinoid granules did not stain.

*Fluorescence.*—The sections were initially examined without prior removal of wax (Barter and Pearse, 1955) but better results were obtained after dewaxing the sections in xylol and mounting them in a non-fluorescent balsam. An intestinal carcinoid which gave positive diazo and argentaffin reactions served as a positive control. A smear preparation of 5-H.T. was also made and after exposing this to formalin vapour (Barter and Pearse, 1955) this gave a similar orange-yellow fluorescence to that of the intestinal carcinoid. Sections of a carcinoma of the cervix and of the stomach served as negative controls. Several sections of the bronchial carcinoid and metastases removed at post mortem did not fluoresce.

*Biological assay.*—Unlike a control solution of 5-H.T., an N/50 HCl extract of a portion of tumour tissue from our case did not cause contraction of a rat uterus or colon.

### *Biochemical Investigations*

For these studies we are greatly indebted to Mr. E. J. Duncan and Professor J. E. Kench of the Department of Chemical Pathology.

The material studied consisted of unfixed tumour, lung and liver tissue obtained post mortem and immediately placed into a deep freeze. Specimens of tumour

---

EXPLANATION OF PLATES

FIG. 1.—The polypoid intrabroncial portion of tumour is seen to be continuous with a larger lobulated mass in the substance of the lung.

FIG. 2.—The tumour cells are arranged in anastomosing columns. × 100.

FIG. 3.—This shows the tumour forming small acinar spaces. × 100.

FIG. 4.—A blood vessel in the lung tumour. The basophilic structures thought to represent nuclear debris can be seen in the grossly thickened walls. × 90.

FIG. 5.—The tricuspid valve. Verhoeff's elastic stain. A loose connective tissue containing elastic fibres has been deposited on the auricular surface of the valve cusp. × 22.

FIG. 6.—This shows the argyrophilic cytoplasmic granules in the tumour cells. × 90.

1

2

3

4

6

5

and of uninvolved liver tissue from a case of gastric carcinoma were used as controls. The tissue was finely ground and extracted with acetone which was evaporated *in vacuo*, and the residue made up to a definite volume with water and defatted with light petroleum. The determination of 5-H.I.A.A. was carried out as described by Udenfriend, Titus and Weisbach (1955) and the figures obtained by this method include all 5-hydroxyindole compounds.

The following results were obtained :

I.—*Case of Carcinoid Syndrome*

| | |
|---|---|
| Uninvolved lung . . | 0.68 mg. per cent |
| Carcinoid tumour in lung . | 2.29 mg. ,, ,, |
| Uninvolved liver tissue . | 0.53 mg. ,, ,, |
| Carcinoid tumour in liver . | 1.91 mg. ,, ,, |

II.—*Control Case*

| | |
|---|---|
| Carcinoma of stomach . | 1.54 mg. ,, ,, |
| Liver tissue . . . | 1.41 mg. ,, ,, |

Extracts of the tumour were also investigated by paper chromatography using a 2-dimensional system—(i) Isopropanol-ammonia, (ii) Butanol-acetic acid. The spots were located by Ehrlich's Aldehyde Reagent.

(1) *Standard solution.*—5-H.T., 3-methylindole (skatole), 3-H.I.A.A., 5-H.I.A.A., tryptophan and urea were used and could be readily separated and identified.

(2) *Uninvolved liver tissue.*—Skatole, 5-H.I.A.A. and urea were present but no 5-H.T. was detected.

(3) *Tumour in Liver.*—5-H.I.A.A., urea, skatole and indole were present. No 5-H.T. could be detected. A red spot (? porphobilinogen) was also noted.

(4) *Uninvolved lung.*—Tryptophan, urea, skatole and indole and a faint trace trace of 5-H.I.A.A. were present.

(5) *Tumour in lung.*—Tryptophan, 5-H.I.A.A. and skatole but no l5-H.T. were present. A red spot (? porphobilinogen) was noted.

Recovery experiments were then performed in which 0·5 mg. of 5-H.T. was added to the tumour and extractions were done as before and investigated chromatographically. When the extract was made immediately after adding the 5-H.T. the presence of 5-H.T., 5-H.I.A.A., skatole, urea and ? porphobilinogen could be demonstrated, but when the extract was made after allowing the tumour and added 5-H.T. to stand at room temperature for 60 min., there was only a faint trace of 5-H.T. and skatole, but no 5-H.I.A.A. nor tryptophan. Similarly, two portions of tumour tissue, each weighing 2 g., were mixed with 0·5 mg. serotonin. One portion was allowed to stand at room temperature for 60 minutes and the other was extracted immediately. In the latter case the recovery was 0·202 mg. (theoretical yield 0·2375 mg. 5-H.I.A.A.) whereas if the extraction was delayed the yield was 0·006 mg.

As a control in the recovery experiments 0·5 mg. 5-H.T. was mixed with portions of the gastric carcinoma used in the earlier estimations and allowed to stand at room temperature before extraction. The theoretical concentration before extraction was 0·2618 mg. per 2 g. of tumour and the true yield was 0·2162 mg. per 2 g. Chromatographic examination of acetone extracts of the gastric carcinoma showed spots of 5-H.I.A.A. and urea. When the tumour was mixed with 0·5 mg. of serotonin and extracted after an hr. at room temperature the spots of 5-H.T., 5-H.I.A.A. and urea were still present.

### DISCUSSION

The purpose of our paper has been to record the autopsy findings in a patient with the clinical and biochemical findings of the carcinoid syndrome and with the primary tumour in the bronchus. The morbid anatomical and histological features of the lung tumour were typical of the carcinoid type of bronchial adenoma and careful examination of the gastro-intestinal tract at autopsy disclosed no primary intestinal carcinoid. The clinical and pathological features of previously reported cases of the carcinoid syndrome due to metastasising bronchial carcinoids have recently been reviewed (Williams and Azzopardi, 1960 ; Weiss and Ingram, 1961) and will not be discussed here.

Certain aspects of our case warrant special comment although their true significance may only emerge when further cases of this type have been studied.

With the exception of the non-specific argyrophilic reaction the histochemical tests in our case were negative in all sections examined. It is well recognised that the argentaffin reaction may be negative in tumours producing the syndrome (Thorson, 1958 ; Waldenström, Pernow and Silwer, 1956), including cases in which the tumour has been found to contain large amounts of 5-H.T. (Smith *et al.*, 1957 ; Thorson, 1958). Therefore it has been postulated that variable staining reactions may, in some cases, be partly related to different secretory activities of the cells rather than autolytic changes. It has not been clear, however, whether the tumours in the cases referred to included cells containing histologically recognisable granules in their cytoplasm. Williams and Azzopardi (1960) have recently suggested that the granules represented a storage product which retains the soluble 5-H.T. in the cytoplasm. It is consequently of considerable interest that in our case, in which the granularity of certain groups of cells was a prominent feature, the specific histochemical tests were negative. The non-reactivity of the cells is furthermore reflected in the failure to demonstrate 5-H.T. by biological and chemical methods. We would therefore suggest that any 5-H.T. originally present in the granules could have disappeared during the long post mortem interval without disturbing the structure of the granules, and thus accounting for their histochemical non-reactivity.

The biochemical studies failed to reveal any 5-H.T. in the tumour tissue, which did, however, contain a greater level of 5-hydroxy-indole compounds than was present in the adjacent univolved tissues. It was of interest that the level of 5-hydroxy-indole compounds was greater in the control specimens than in the tumour-free lung and liver from our case. The post mortem intervals were similar in the two cases. Possibly related to this finding is the apparent disappearance of 5-H.T. added to the carcinoid tumour *in vitro*, whereas this did not occur when 5-H.T. was added to the gastric carcinoma. It must remain conjectural whether or not this represents a true enzymatic destruction of the 5-H.T.

Of the four recorded autopsy cases in which the carcinoid syndrome was associated with a primary bronchial tumour only that of Weiss and Ingram (1961)

showed the typical cardiac abnormality. The tricuspid valve lesions in our case corresponded to those described in some cases by Thorson (1958) in that a loose collagenous tissue had been superimposed on a cusp which itself was myxomatous but elsewhere contained an increased amount of collagen. The pulmonary valve changes, consisting of a collagenous tissue lying superficial to the elastic lamina of a relatively normal cusp, are similar to those described by MacDonald and Robbins (1957) and to the superficial fibrous pad mentioned by Thorson (1958), and may represent an earlier lesion than that seen in the tricuspid valve. Although Smith and Campbell (1956) suggested that the valves on the left side of the heart may be involved more frequently than is generally recognised, the assessment of these alterations is complicated by the fact that these valves not infrequently show non-specific nodular thickenings in autopsies on adult patients, and the changes we have described may well be of this type.

The changes in the thyroid resembling Hashimoto's disease and the cirrhosis are of interest in view of the fibrogenic properties of serotonin. The literature contains very few references to the thyroid, and although fatty change of the liver and cirrhosis have rarely been recorded (MacDonald, 1956 ; Zarafonetis, Lorber and Hanson, 1958), their significance is uncertain. A further complicating factor in our case is that the liver had been irradiated, and although the deep scar along the anterior border of the liver appeared to be secondary to spontaneous or irradiation induced necrosis of tumour, it would be difficult to account for the more diffuse fibrosis elsewhere on this basis.

The hyaline thickening of the pulmonary arteries in our case is worthy of mention as similar changes were described by Hand, McCormick and Lumb (1958). Although noted in both lungs, they were more prominent on the left side. An irradiation effect therefore had to be considered, but there were no other signs of a radiation pneumonitis. Furthermore, we have observed similar though less pronounced hyaline thickenings of small pulmonary arteries in autopsies on middle-aged subjects, and the significance of these changes must remain open to question. Bone metastases have only infrequently been recorded in metastasising intestinal or bronchial carcinoids. Toomey and Felson (1960) drew attention to the usual osteosclerotic nature of the bony secondaries and this feature was also seen in our case.

Radiation therapy of carcinoid tumours has received scant attention but Pearson and Fitzgerald (1949) refer to isolated reports suggesting that the tumours were radiosensitive. Irradiation of the liver resulted in temporary symptomatic relief in our patient and similar treatment to the spine relieved her sciatica.

### SUMMARY

The autopsy findings have been recorded in a patient who presented the clinical and biochemical features of the carcinoid syndrome in association with a metastasising bronchial adenoma.

Failure to demonstrate 5-hydroxytryptamine in the tumour or its metastases by histochemical methods was reflected in an absence of chromatographically or biologically detectable amounts of the substance.

Attention has been drawn to the presence of cirrhosis of the liver and to changes resembling those of Hashimoto's disease in the thyroid gland.

### REFERENCES

ADAMS, C. W. M.—(1957) *J. clin. Path.*, **10**, 56.
BARTER, R. AND PEARSE, A. G. E.—(1955) *J. Path. Bact.*, **69**, 25.
BODIAN, D.—(1936) *Anat. Rec.*, **65**, 89.
HAND, A. M., McCORMICK, W. F. AND LUMB, G.—(1958) *Amer. J. clin. Path.*, **30**, 47.
KRIKLER, D. M., LACKNER, H. AND SEALY, R.—(1958) *S. Afr. med. J.*, **32**, 514.
LILLIE, R. D. AND GLENNER, G. G.—(1960) *Amer. J. Path.*, **36**, 623.
MACDONALD, R. A.—(1956) *Amer. J. Med.*, **21**, 871.
*Idem* AND ROBBINS, S. L.—(1957) *Arch. Path.*, **63**, 103.
PEARSE, A. G. E.—(1960) ' Histochemistry ', 2nd Edition. London (J. & A. Churchill).
PEARSON, C. M. AND FITZGERALD, P. J.—(1949) *Cancer*, **2**, 1005.
SMITH, A. N., NYHUS, L. M., DALGLEISH, C. E., DUTTON, R. W. AND MACFARLANE, P. S. —(1957) *Scot. med. J.*, **2**, 24.
SMITH, J. P. AND CAMPBELL, A. C. P.—(1956) *J. Path. Bact.*, **72**, 673.
THORSON, A. H.—(1958) *Acta med. scand.*, Vol. **161**, Suppl. 334, 1.
TOOMEY, F. B. AND FELSON, B.—(1960) *Amer. J. Roentgenol.*, **83**, 709.
UDENFRIEND, S., TITUS, E. AND WEISBACH, H.—(1955) *J. biol. Chem.*, **216**, 499.
WALDENSTRÖM, J., PERNOW, B. AND SILWER, H.—(1956) *Acta med. scand.*, **156**, 73.
WEISS, L. AND INGRAM, M.—(1961) *Cancer*, **14**, 161.
WILLIAMS, E. D. AND AZZOPARDI, J. G.—(1960) *Thorax*, **15**, 30.
ZARAFONETIS, C. J. D., LORBER, S. H. AND HANSON, S. M.—(1958) *Amer. J. med. Sci.*, **236**, 1.